# Effect of Endurance and Strength Training on the Slow Component of 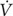
*O*_2_ Kinetics in Elderly Humans

**DOI:** 10.3389/fphys.2018.01353

**Published:** 2018-10-09

**Authors:** Enrico Tam, Paolo Bruseghini, Carlo Capelli, Eugenio Oliboni, Andrea Pezzato, Silvia Pogliaghi, Roberto Pozzi Mucelli, Federico Schena, Elisa Calabria

**Affiliations:** ^1^Department of Neurological and Movement Sciences, School of Sport and Exercise Sciences, University of Verona, Verona, Italy; ^2^Department of Molecular and Translational Medicine, University of Brescia, Brescia, Italy; ^3^Department of Physical Performances, Norwegian School of Sport Sciences, Oslo, Norway; ^4^Institute of Radiology, School of Medicine, Policlinico “GB Rossi”, Department of Pathology and Diagnostics, School of Medicine, University of Verona, Verona, Italy

**Keywords:** high intensity interval training, isoinertial strength training, heavy intensity exercise, near-infrared spectroscopy, oxygen uptake kinetics, elderly, muscle strength, slow component

## Abstract

We compared the effects of 8 weeks of high intensity, aerobic interval training (*HIT)* and isoinertial resistance training (*IRT)* on: (i) 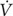
*O*_2_ kinetics during heavy (*HiEx*) intensity exercise and; (ii) work economy during moderate (*ModEx*) intensity exercise in 12 healthy elderly men (69.3 ± 4.2 years). Breath-by-breath 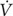
*O*_2_ and muscle deoxygenation ([*HHb*] by means of *NIRS*) were measured in *HiEx* and *ModEx* at identical workloads before and after trainings. In *HiEx*, 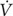
*O*_2_ and *HHb* responses were modeled as tri-exponential and mono-exponential increasing functions, respectively. A two-way ANOVA for repeated measures analysis was made; Effect size (η^2^) was also evaluated. After *HIT* the amplitude and the time delay of the slow component of *O_2_* uptake (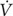
*O*_2sc_) during *HiEx* were smaller (−32%; *P* = 0.045) and longer (+19.5%; *P* = 0.001), respectively. At *Post IRT*: (i) during *ModEx, gain* was lower (−5%; *P* = 0.050); (ii) during *HiEx*, τ_2_ (+14.4%; *P* = 0.050), *d_3_* (+8.6%; *P* = 0.050), and τ_3_ (+17.2%; *P* = 0.050) were longer than at *Pre IRT*. After *HIT*, the decrease of the 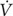
*O*_2sc_ amplitude was likely induced by the beneficial effects of training on a more responsive *O*_2_ delivery and consumption cascade leading to a better muscle metabolic stability. *IRT* training was able to increase exercise economy during *ModEx* and to reduce the amplitude and delay the onset of 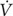
*O*_2sc_ during *HiEx*. These effects should be due to the reduction and the delayed recruitment of Type II muscle fibers. The better exercise economy and the delayed appearance of 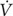
*O*_2sc_ induced by *IRT* suggests that strength training might be included in endurance training programs to improve exercise economy and resistance to fatigue in this population of old subjects.

## Introduction

The kinetics of alveolar *O*_2_ uptake (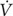
*O*_2A_) upon the onset of constant work rate (*CWR*) exercise of moderate intensity (*ModEx*) is usually described by a double exponential model (Poole and Jones, 2012). The first, rapid component – phase I – is characterized by a short time constant and it is caused by the prompt increase of cardiac output at the beginning of exercise. The second component – phase II – is considered to be a reliable proxy of muscular *O*_2_ uptake and is characterized by a time constant of about 20 s in young, healthy, and trained subjects (Poole and Jones, 2012).

During heavy intensity exercise (*HiEx*), i.e., above the lactic threshold (*LT*), the attainment of the steady state oxygen consumption (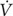
*O*_2ss_) is delayed due to the presence of a slow increase of 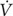
*O*_2_(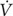
*O*_2sc_) that starts about 150–200 s after the onset of exercise (Jones et al., 2011). Furthermore, if the exercise is performed in the very heavy domain (*VHiEx*), e.g., above the so called critical power, 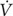
*O*_2ss_ cannot even be attained, since 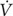
*O*_2_ keeps increasing up to 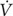
*O*_2max_, a condition that heralds the interruption of exercise (Poole and Jones, 2012).

From the performance standpoint, 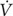
*O*_2sc_ is important, as it is related to increased susceptibility to fatigue: 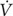
*O*_2sc_ amplitude, e.g., is linearly related to the time to fatigue in obese adolescents (Salvadego et al., 2010).

There is compelling evidence that muscular mechanisms are largely responsible for 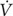
*O*_2sc_ (Poole et al., 1991) and several data support the notion that the progressive recruitment of Type II muscle fibers during *HiEx/VHiEx* exercise is the main determinant of 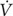
*O*_2sc_ (Poole and Jones, 2012). Type II fibers are characterized by a higher *ATP* cost of force production (Stienen et al., 1996) and by higher *O*_2_ consumption for *ATP* synthesis (Willis and Jackman, 1994) than Type I fibers and it has been also demonstrated that 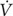
*O*_2sc_ is more evident in humans with a higher percentage of Type II fibers (Barstow et al., 1996). Recent findings, however, have somehow challenged this view suggesting that the progressive recruitment of the less economic Type II fibers is not strictly necessary to induce 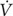
*O*_2sc_. Conversely, 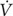
*O*_2sc_ may be caused by events occurring inside the recruited fibers (Zoladz et al., 2008).

In addition, it has been shown that 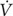
*O*_2sc_ can be modulated by manipulations of *O*_2_ delivery (Poole and Jones, 2012). Therefore, decreased *O*_2_ availability may affect the 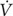
*O*_2sc_ of individuals in whom local *O*_2_ delivery during exercise is impaired (e.g., healthy aging) and a clear mismatch between *O*_2_ delivery and consumption is present (Murias et al., 2010a,b).

The effects of physical training have been explored to disclose the mechanisms underpinning 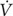
*O*_2sc_ (Jones et al., 2007). Endurance training improves the so-called metabolic stability, leading to a lower decrease in phosphocreatine concentration *[PCr]* and a diminished intramuscular acidosis during *HiEx* in connection with a less evident 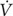
*O*_2sc_ (Poole and Jones, 2012). Since low levels of intramuscular *[PCr]* and of *pH* characterize *HiEx/VHEx* exercise (Jones et al., 2008, 2011), these results seem to suggest that the slow decrease in *[PCr]* and increase of *[H^+^]* occurring at these exercise intensities (Jones et al., 2008) are the main mechanistic determinants of 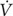
*O*_2sc_. In addition, endurance training improves metabolic hyperemic response and optimizes the matching between local *O*_2_ delivery and utilization, especially in individuals with suboptimal vascular response, such as elderly subjects (Murias et al., 2010a,b). Therefore, the correlation between the indexes that describe amelioration of local peripheral perfusion and the attenuation of the amplitude of 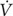
*O*_2sc_ might suggest a potential mechanistic link between *O*_2_ delivery and 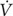
*O*_2sc_.

Also, strength training, by decreasing the number of motor units (MUs) recruited at the same work rate (*WR)*, may theoretically attenuate 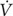
*O*_2sc_, as a smaller number of less economic Type II fibers would be recruited at the same *WR*. However, this hypothesis has been somehow disproved in young adults in whom isometric strength training failed to abate the amplitude of 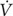
*O*_2sc_ (Zoladz et al., 2012). Yet, more effective strength training modalities applied to subjects with large muscular strength deficits may potentially elicit more evident and beneficial effects on 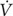
*O*_2sc_ via this mechanism.

Finally, it has also been suggested that strength training may improve mechanical efficiency during *ModEx* (Beattie et al., 2014). From the practical standpoint, a greater exercise economy associated with the attenuation of 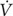
*O*_2sc_ induced by strength training may ameliorate exercise capability in subjects characterized by a low exercise capacity.

Therefore, we studied in a group of healthy, moderately active elderly men the effect of high intensity interval training (HIT) and isoinertial strength training (*IRT)* on: (1) 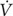
*O*_2_ kinetics and muscular oxygenation of the exercising muscle by near-infrared spectroscopy (*NIRS*) during cycling *HiEx* performed at the same absolute *WR* before and after training; (2) work economy during *ModEx* at the same absolute WR. In addition, (3) Muscle cross sectional area (*CSA*) and muscle volume (*Vol*) of the quadriceps; and (4) muscular strength were assessed. We analyzed these data to determine the effects and relative mechanisms induced by *HIT* and *IRT* on the entity of 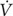
*O*_2sc_.

## Materials and Methods

### Subjects

Twelve moderately active Caucasian men (mean ± SD; 69.3 ± 4.2 years, range, 65–75; 77.8 ± 10.4 kg; height 1.72 ± 0.05 m) volunteered to participate in the study. A medical examination, to determine exclusion criteria, and a cycle-ergometer stress test, to exclude abnormal responses to intense exercise, were preliminarily performed. The study protocol was approved by the institutional review board (approval on June 18th, 2013) and designed in accordance with ethical standards, the provisions of the Declaration of Helsinki and national and international guidelines. Written informed consent was obtained from each subject before the study.

### Experimental Design

**FIGURE 1:**
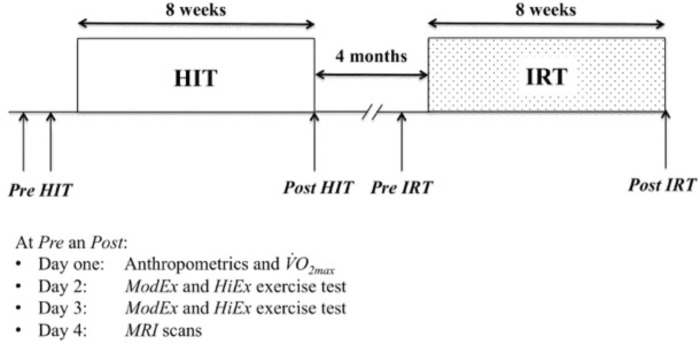
Schematic representation of the experimental design.

A two-factor within-subject design (A × B × S) (Keppel and Wickens, 2004) was used in which each subject (factor, S) received all the combinations that originated by crossing the two factors A and B. One fixed factor (A) was training modality (levels: *HIT* and *IRT*); the second fixed factor (B) was time (levels: *Pre* and *Post* training). The subjects were evaluated immediately before (*Pre HIT*) and immediately after 8 weeks of *HIT* (*Post HIT*). Then, after 4 months of recovery during which the subjects were asked to keep the same habitual lifestyle (**Figure 1**), the subjects were evaluated again before (*Pre IRT*) and immediately after 8 weeks of *IRT* (*Post IRT*). Before the first data collections, a familiarization session was conducted.

*MRI* scans for measuring muscle *CSA* and *Vol* were obtained before and after *HIT* and *IRT*.

Tests were performed in the morning on four consecutive days: the main anthropometrical data and 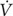
*O*_2 max_ were measured on day 1; *CWR ModEx* and *HiEx* exercise tests were performed on days 2 and 3; *MRI* scans were obtained on day 4.

### Training Protocols

• *High intensity interval training (HIT).* The subjects trained three times a week for 8 weeks. Training consisted of seven 2-min bouts of cycling (915 E, Monark, Varberg, Sweden) at about 85–95% of individual 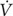
*O*_2 max_ interspersed by 2 min of recovery at about 40% of 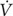
*O*_2 max_. Each series was preceded by 10 min of active warm-up.
• *Isoinertial resistance training (IRT).* Resistance exercise was performed on a seated knee extension flywheel (4.2 kg) ergometer (YoYo Technology AB, Stockholm, Sweden) three times a week for 8 weeks. Each session consisted of four sets of seven maximal, coupled concentric extensions and eccentric flexions of the knee. The sets were interspersed by 3-min of rest and initiated immediately after performing two submaximal actions. Each exercise session was preceded by 10 min of active warm-up.

### Anthropometry

Body weight (*BW*) and stature were measured with a Tanita electronic scale BWB-800 MA (Tanita, Arlington Heights, IL, United States) and a stadiometer (Holtain Ltd., Crymych, Pembs. United Kingdom).

### Maximal Oxygen Uptake, Ventilatory Thresholds

All cycling tests were performed on an electromechanically braked cycle ergometer (Excalibur Sport, Lode, Netherlands) operated by a personal computer connected to a metabolic cart. Breath-by-breath gas exchanges were measured continuously at the mouth with a metabolic cart (Quark b^2^, Cosmed, Rome, Italy) that was calibrated following the manufacturer’s instructions before each experiment.


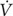
*O*_2 max_ and ventilatory thresholds were measured during a ramp test (Poole et al., 2008) and a supra maximal *CWR* test following the procedure illustrated by Bruseghini et al. (2015).

### Responses to Moderate Intensity and Heavy-Intensity Exercise

Responses to *ModEx* and *HiEx* exercise were evaluated at a *WR* corresponding to 90% of individual gas exchange threshold (*GET*) and to about 50% of the difference between *GET* and respiratory compensation point (*RCP*) determined at *Pre HIT*. The *WR* was calculated using the linear regression of 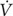
*O*_2_ vs. *WR* considering the lag of the 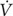
*O*_2_ increase with respect to that of the workload determined at the ramp test and it was maintained constant in all the sessions. After instrumentation and preparation, the subjects rested on the cycle ergometer for 3 min before starting to pedal for 3 min at 30 W; then, *WR* was increased to the preselected *WR* and maintained for 6 min. The procedure was repeated three times (twice for *ModEx* and once for *HiEx*) with 10 min of recovery between each test. The entire procedure was repeated the following day. Pedaling frequency was strictly maintained between 70 and 80 revolutions per minute by the aid of a visual pacemaker.

### Muscle Oxygenation

Vastus lateralis muscle oxygenation during *HiEx* was evaluated by means of a frequency-domain-multidistance *NIRS* system (OxiplexTS, ISS, Champaign, IL, United States) that provided continuous measurement of absolute concentrations (μM) of oxyhemoglobin (*[O_2_Hb]*) and deoxyhemoglobin (*[HHb]*) (De Roia et al., 2012). The thickness of the skin and of the subcutaneous fat layer of the explored area was assessed by ultrasound (ACUSON P50 ultrasound system, Siemens, Erlangen, Germany) and it ranged from 6.4 to 11.8 mm, 8.1 mm ± 1.5. In addition, cutaneous landmarks were pen-marked on a transparent acetate sheet placed on the area of the probe so that it could be applied on the same site in the subsequent experimental sessions.

### Muscular Strength and Morphology

Knee extension torque (*T*_k_) of the dominant limb was evaluated with an isokinetic dynamometer (CMSi Cybex Humac Norm Dynamometer, Stoughton, MA, United States) during concentric contractions at 60° s^−1^ and 120° s^−1^ angular speeds. The subjects went through several practice trials and performed contractions while seated on the reclining chair of the dynamometer. The lower part of the leg was strapped to the end of the lever arm and the center of rotation of the knee was aligned with the axis of the dynamometer. Before the test, the subjects completed 10-min of warm-up exercise on a stationary bike. Three maximal trials were performed for each condition with 3 min of recovery between each trial. The highest *T*_k_ values (as peak values) were recorded for further analysis.

*MRI* scans were obtained after 1 h of supine rest to avoid the influence of posture-related fluid shifts on muscle size following the procedure illustrated by Bruseghini et al. (2015).

### Data Analysis

*Breath-by-breath* 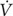
*O*_2_ values were interpolated to 1 s intervals, time aligned with the onset of exercise transition, and treated by subtracting the 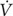
*O*_2_ steady state value (average values of the last 30 s of trial) at 30 W. The data from the trials were then combined to obtain a single data file for each subject and condition.


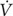
*O*_2_ kinetics during *HiEx* exercise was modeled as a sum of three exponential increasing functions:

(1) $$\begin{array}{l} \dot{V}O_{2}=U(t-d_{1})\times (A_{1}\times (1-e^{-(t-d_{1}/\tau_{1})}))+\\ \;U(t-d_{2})\times (A_{2}\times (1-e^{-(t-d_{2}/\tau_{2})}))+\\ \;U(t-d_{3})\times (A_{3}\times (1-e^{-(t-d_{3}/\tau_{3})})) \end{array}$$

where τ_1_, τ_2_, and τ_3_ are the time constants of the exponential increases during phase I, phase II, and phase III (the slow component), *d*_1_, *d*_2_, and *d*_3_ are the time delays and *A*_1_, *A*_2_, and *A*_3_ are the asymptotic amplitudes of the corresponding phases. *U (t − d)* is the unit step function defined as:

(2) $$U(t-d)\{\frac{0\;\mathrm{if}\;t<d}{1\;\mathrm{if}\;t\geq d}$$

The value of the amplitude at the end of phase I, (*A*′_1_), which terminated at the start of phase II, was calculated as (2):

(3) $${A^{\prime }}_{1}=A_{1}\times \left(1-e^{-(d_{2}/\tau_{1})}\right)$$

Note that the first addend of Eq. (1) is truncated as it reaches *A*′_1_ at *t* = *d*_2_, and doesn’t continue to rise toward its asymptotic value *A*_1_. The physiologically significant amplitude of the primary exponential (*A*′_2_) was defined as the sum of *A*′_1_ + *A*_2_ (Barstow et al., 1996). Because of the uncertain validity of the asymptotic value of *A*_3_, we used the value of the amplitude of the slow component at the end of the exercise (*A*′_3_) (Barstow et al., 1996). The change of *O*_2_ uptake from the 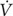
*O*_2_ steady state value at 30 W and the values of 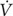
*O*_2_ at *A*′_3_ (Δ
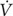
*O*_2_*EE*) was given by *A*′_2_ + *A*′_3_. To compare the subjects working at different absolute workloads, the gain in the primary response (*G*_Prim_ = *A*′_2_/ΔWR) and the gain in the total response at the end of *HiEx* exercise [*G*_Tot_ = (*A*′_2_ + *A*′_3_)/ΔWR] were calculated. The relative contribution of the slow component to the overall 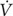
*O*_2_ response was calculated as *A*′_3_/(*A*′_2_ + *A*′_3_).

The gain (*G*) during *ModEx* exercise was calculated as the ratio between net steady state 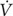
*O*_2_ and the corresponding net increase of WR (*G* = *A*′_2_/ΔWR).

*NIRS* derived *[HHb]* response during *HiEx* was first interpolated to 1-s intervals, then time aligned with the onset of exercise transition and finally treated by subtracting the steady state value at 30 W. Then, the fitting window was constrained from the start of exercise to the onset of the slow component of *[HHb]* (Breese et al., 2013). Mean response time (*MRT*) was calculated as the sum of τ_1_ and *d*_1_. The primary *[HHb]* amplitude was divided by the phase II asymptotic amplitude *A_2_* to yield the Δ*[HHb]*/Δ
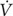
*O*_2_: it was considered as an index of the increase in fractional muscle *O*_2_ extraction required to sustain a given net increment in 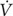
*O*_2_ during the primary phase (Murias et al., 2014). The net increase from the baseline of the values of *[HHb]* and of *[O*_2_*Hb]* after 120 s of exercise and at the end of the exercise were calculated over 30 s time windows, the first interval of time being centered on the 120th-second and second interval including the last 30 s of exercise.

The net increases in *[HHb]* and in *[O*_2_*Hb]* were then added to obtain the net increase in total hemoglobin concentration (*[Hb*_tot_*]*) in the volume of tissue explored by the probe. *[Hb*_tot_*]* was only calculated at 120 s of exercise and at the end of exercise.

The parameters of the 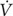
*O*_2_ models were estimated by means of an iterative, weighted non-linear least-squares procedure (Marquardt, 1963) that was developed in G-Language (Lab-VIEW 7.0, National Instruments, Austin, TX, United States). Initial guesses of the parameters of the model were entered after visual inspection of the data. The 95% confidence intervals of the τ_2_ and τ_3_ of 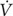
*O*_2_ kinetics and of τ_1_ of *HHb* kinetics were generated by means of Monte Carlo simulation (Motulsky and Christopoulos, 2004) using commercial software for data analysis (GraphPad Prism version 6.00 for Macintosh, GraphPad Software, La Jolla, CA, United States). Amplitudes and time delays were constrained to the best-fit values and the time constants were allowed to vary.

*MRI* scans were transferred electronically from the scanner to a personal computer (Macintosh mac Book Pro, Apple, Cupertino, CA, United States) and analyzed with OsiriX (version 3.7.1 32 bit) by using manual planimetry to calculate *CSA* and *Vol* of the quadriceps of the dominant leg (Bruseghini et al., 2015). The same investigator carried out all measurements. The reliability of this measurement was assessed over five separate measurements of the *CSA* of three heads of the quadriceps muscle taken distally at 50% of the femur bone length; the average coefficient of variation of measuring the same image was 0.92% for total *quadriceps femoris*.

### Statistical Analysis

All values in the text and the tables are presented as mean ± SD. Two-factor within-subject ANOVA analysis for repeated measures was carried out according to Keppel and Wickens (2004): (i) *F* values were calculated taking into account the possible violation of sphericity as suggested by Geisser and Greenhouse; (ii) single contrasts within subjects (time, *Pre* vs. *Post*) and between subjects (Training, *HIT* vs. *IRT* and interactions were computed; (iii) effect size was evaluated with partial squared correlation factor or η^2^, ($\eta_{w}^{2}$, $\eta_{b}^{2}$, $\eta_{\mathrm{inte}}^{2}$, suffix are related to within, between, and interactions analysis) which expresses the ratio between explained variability and total variability in the population, but compensates for the size of the other treatment effect (either time or training); (iv) effect size (*d*) of the differences between the contrasted values was calculated. Calculations were carried out using an Excel spreadsheet (MO 2010, Microsoft Corp., Seattle, WA, United States) prepared for this purpose. Model 2 linear regressions between bivariate data were calculated according to the method of Deming (Motulsky and Christopoulos, 2004). Correlation between variables was computed using Spearman’s correlation coefficient.

Statistical analysis was made by a two-way ANOVA for repeated measures; Effect size was evaluated with partial squared correlation factor or η^2^. *P* was always set <0.05.

## Results

The data concerning 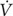
*O*_2 max_, ventilatory threshold and muscular strength and mass have been already published in a paper that described the effects of *HIT* and *IRT* on several risk factors of cardiometabolic diseases and on the exercise capability in healthy elderly subjects (Bruseghini et al., 2015). The readers are kindly asked to refer to the indicated paper for further details. Here, only the essential results useful for supporting and discussing the hypothesis related to the present investigation will be summarized.

Briefly, absolute 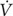
*O*_2 max_ increased only after *HIT* (*Pre HIT* 2.34 ± 0.35 *Post HIT* 2.48 ± 0.38 L min^−1^ *P* = 0.015; *d* = 0.83; 95% *CI*_Diff_: 0.04 L min^−1^/0.22 L min^−1^), with no differences after IRT (*Pre IRT* 2.43 ± 0.43 *Post IRT* 2.44 ± 0.42 L min^−1^). 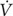
*O*_2RCP_, expressed as percent of 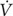
*O*_2 max_, was greater at *Post HIT* (*P* = 0.014; *d* = 0.85; 95% *CI*_Diff_: 2.1%/11.1%) and at *Pre IRT* (*P* = 0.007; *d* = 0.96; 95% *CI*_Diff_: 3.9%/16.2%) than at *Pre HIT* and it was greater at *Post IRT* than at *Post HIT* (*P* = 0.001; *d* = 1.24; 95% *CI*_Diff_: 1.4%/3.8%). *Post hoc* contrast analysis showed that *CSA* and *Vol* were increased after *HIT*: *Vol*, *Pre HIT*: 820 ± 199 cm^3^, *post HIT*: 866 ± 199 cm^2^; *P* = 0.002; *d* = 1.17; 95% *CI*_Diff_: 22.9 cm^3^/67.9 cm^3^) and after *IRT Vol*, *Pre IRT*: 813 ± 184 cm^3^, *post IRT*: 852 ± 188 cm^2^; *P* = 0.01; *d* = 0.90; 95% *CI*_Diff_: 13.9 cm^3^/64.6 cm^3^). Finally, maximal isokinetic torque was increased only after *IRT*: *T*_k_ *60° s*^−1^, *Pre HIT*: 159.8 ± 24.5 N m, *post HIT*: 163.3 ± 22.2 N m; *P* = 0.360; *d* = 0.27; 95% *CI*_Diff_: −3.9 N m/10.9 N m; *T*_k_ *60° s*^−1^, *Pre IRT*: 162.4 ± 25.8 N m, *post IRT*: 179.0 ± 31.1 N m; *P* = 0.001; *d* = 1.27; 95% *CI*_Diff_: 9.0 N m/24.1 N m.

### Response to *ModEx*

The average *CWR* was 72.5 ± 16.3 W in the *ModEx* condition, corresponding to 35–40% of 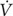
*O*_2 max_, i.e., <*GET*. *G* at *Pre HIT* and at *Post HIT* was not significantly different (12.1 mL min^−1^ W^−1^± 1.5 vs. 12.4 mL min^−1^ W^−1^± 1.0). Conversely, at *Post IRT* (12.0 mL min^−1^ W^−1^± 1.0) *G* turned out to be significantly smaller (*P* = 0.049; *d* = 0.63; 95% *CI*_Diff_: −0.05 mL min^−1^ W^−1^/−1.1 mL min^−1^ W^−1^) than at *Pre IRT* (12.6 mL min^−1^ W^−1^± 0.9).

### Response to *HiEx*

**FIGURE 2:**
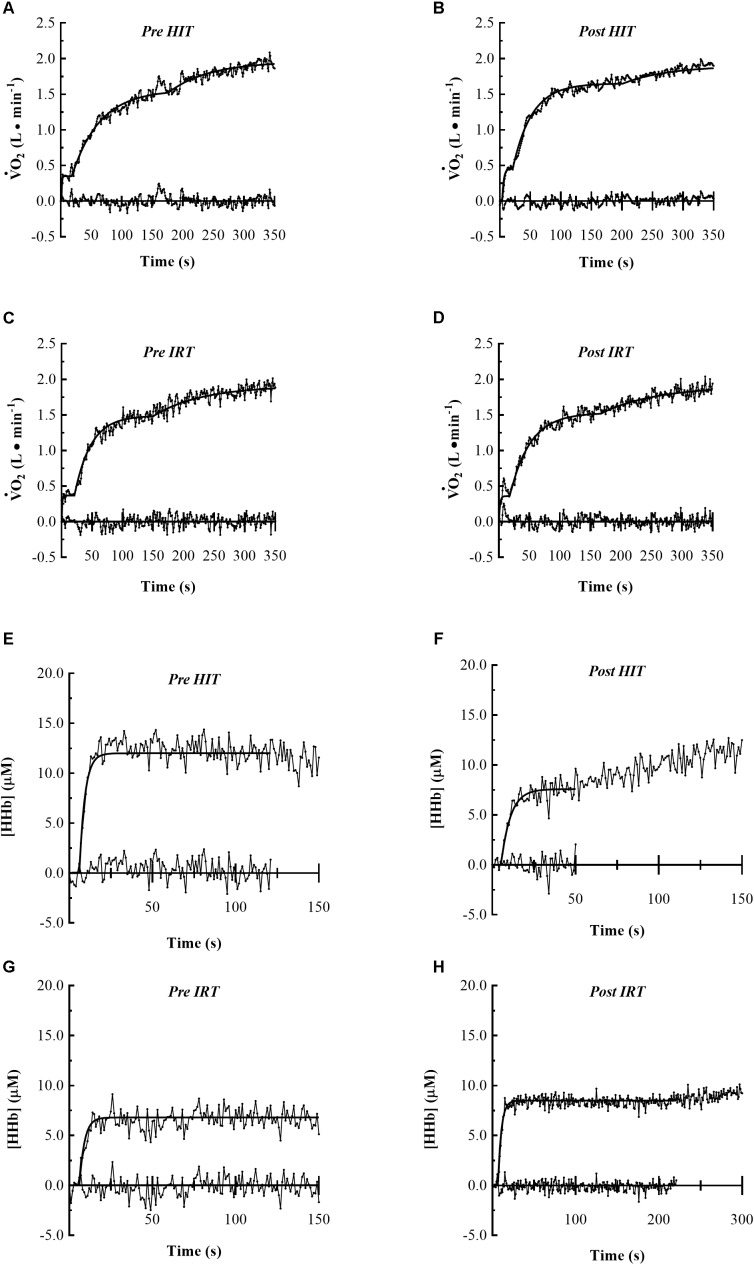
Pulmonary 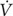
*O*_2_ and muscle *HHb* kinetics of typical subjects at the onset of constant work rate exercise of heavy intensity are represented. The first four panels show the following 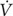
*O*_2_ kinetics: *Pre HIT* **(A)**, *Post HIT* **(B)**, *Pre IRT* **(C)** and *Post IRT* **(D)**. The last four panels show the muscle *HHb* kinetics: *Pre HIT* **(E)**, *Post HIT* **(F)**, *Pre IRT* **(G)** and *Post IRT* **(H)**. Data are displayed on 1 s base and the residual plot is shown on *x*-axis.

The average *CWR* was 144.3 ± 26.6 W in the *HiEx* condition and it corresponded approximately to 67–71% of 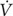
*O*_2 max_, i.e., >*GET*, but <*RCP*. The parameters describing the kinetics of 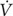
*O*_2_ and the *NIRS* signals obtained in the *HiEx* condition before and after *HIT* and *IRT* are presented in **Tables 1**, **2**, respectively; **Figures 2A–D** demonstrates the kinetics of 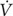
*O*_2_ at the onset of *HiEx* after *HIT* and *IRT* in a typical subject, respectively. **Figures 2E–H** shows the kinetics of *[HHb]* after *HIT* and *IRT*, respectively.

**Table 1:** Mean values (SD) of the parameters describing 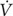
*O*_2_ kinetics at the onset of *CWR* exercise of heavy (*HiEx*) intensity.

Parameter				Training			
				HIT		IRT	
	*P*_b_; $\eta_{b}^{2}$	*P*_w_; $\eta_{w}^{2}$	*P*_int_; $\eta_{\mathrm{inter}}^{2}$	Pre	Post	Pre	Post
*A*’_1_ (L min^−1^)	0.061; 0.271	0.098; 0.233	0.248; 0.123	0.37 (0.15)	0.37 (0.20)	0.23^∗^ (0.18)	0.35^‡^ (0.10)
τ_1_ (s)	0.006; 0.508	0.051; 0.305	0.535; 0.036	2.1 (1.5)	3.9 (3.2)	1.0^∗^ (0.7)	2.0 (1.9)
*d*_1_ (s)	0.024; 0.341	0.213; 0.136	0.264; 0.112	1.3 (1.5)	2.3 (2.7)	0.5 (1.1)	0.5^†^ (0.5)
*A*’_2_ (L min^−1^)	0.101; 0.227	0.025; 0.381	0.751; 0.009	1.45 (0.27)	1.53 (0.22)	1.51 (0.22)	1.58^‡,^(0.34)
τ_2_ (s)	0.227; 0.131	0.410; 0.063	0.013; 0.444	27.7 (7.0)	24.9 (4.3)	25.7 (3.7)	30.0^‡,†^ (5.1)
95% *IC* τ_2_ (s)				26.1–29.4	23.9–25.9	24.2–27.1	27.9–30.9
*d*_2_ (s)	0.006; 0.508	0.249; 0.119	0.687; 0.015	17.7 (3.1)	19.3 (8.7)	12.1^∗^ (5.0)	14.9 (3.8)
*A*’_3_ (L min^−1^)	0.328; 0.088	0.019; 0.407	0.844; 0.004	0.19 (0.10)	0.13^∗^ (0.07)	0.21 (0.12)	0.16 (0.10)
τ_3_ (s)	0.003; 0.570	0.411; 0.062	0.127; 0.199	71.0 (8.5)	69.6 (18.4)	81.5 (16.7)	92.0^†^ (15.6)
95% *IC* τ_3_ (s)				70.3–71.8	69.4–71.2	78.6–81.7	90.0–94.7
*d*_3_ (s)	0.851; 0.003	0.000; 0.742	0.047; 0.313	165.8 (10.1)	197.1^∗^ (13.6)	172.2 (43.2)	185.8^‡^ (42.6)
Δ 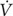 *O*_2EE_ (L min^−1^)	0.015; 0.433	0.398; 0.07	0.665; 0.018	1.62 (0.30)	1.67 (0.27)	1.72 (0.27)	1.74 (0.34)
*A*’_3_/(*A*’_2_ + *A*’_3_) (%)	0.329; 0.067	0.010; 0.471	0.706; 0.013	13.8 (7.5)	8.5^∗^ (3.8)	14.8 (9.5)	10.8 (6.6)
*G*_Prim_ (mL min^−1^ΔW)	0.081; 0.251	0.040; 0.317	0.626; 0.012	10.1 (1.3)	10.7 (0.5)	10.5 (0.9)	10.9 (0.8)
*G*_Tot_ (mL min^−1^/W)	0.061; 0.292	0.637; 0.024	0.954; 0.000	11.5 (1.5)	11.6 (0.6)	12.3 (1.5)	12.4 (1.5)

*The table reports also the mean of the 2.5% and 97.5% percentiles of the 95% confidence interval for τ_2_ and the P-values of the ANOVA analysis together with the values of the corresponding partial squared correlation factors. For the meaning of the symbols, please refer to the text. ^∗^Significantly different from Pre HIT; ^†^significantly different from Post HIT; ^‡^significantly different from Pre IRT.*

The amplitude *A*′_1_ of phase I at *Post IRT* was significantly larger than at *Pre IRT* (*P* = 0.018; *d* = 0.81; 95% *CI*_Diff_: 0.03 L min^−1^/0.20 L min^−1^); the latter value was also significantly smaller than at *Pre HIT* (*P* = 0.029; *d* = 0.72; 95% *CI*_Diff_: 0.03 L min^−1^/0.24 L min^−1^). The time delay in phase I at *Post IRT* was significantly shorter than at *Post HIT* (*P* = 0.036; *d* = 0.69; 95% *CI*_Diff_: −0.3 s/−2.3 s) (**Table 1**).

*A*′_2_ was greater at *Post IRT* than at *Pre IRT* (*P* = 0.028; *d* = 0.73; 95% *CI*_Diff_: 0.01 L min^−1^/0.12 L min^−1^). The time constant of the primary phase of 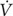
*O*_2_ kinetics (τ_2_) during *HiEx* was significantly longer at *Post IRT* than before strength training (*P* = 0.010; *d* = 0.90; 95% *CI*_Diff_: 1.5 s/6.9 s) and after *HIT* (*P* = 0.010; *d* = 1.02; 95% *CI*_Diff_: 2.2 s/7.9 s). In addition, a significant interaction between training types and time at τ_2_ was noted (*P* = 0.010; *d* = 0.94; 95% *CI*_Diff_: 2.6 s/13.2 s). This further suggests that *IRT* was specifically able to induce the deceleration of the primary phase of 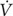
*O*_2_ kinetics during *HiEx*. Finally, *d_2_* at *Pre IRT* was shorter than before *HIT* (*P* = 0.004; *d* = 1.04; 95% *CI*_Diff_: −2.5 s/−8.8 s) (**Table 1**).

**FIGURE 3:**
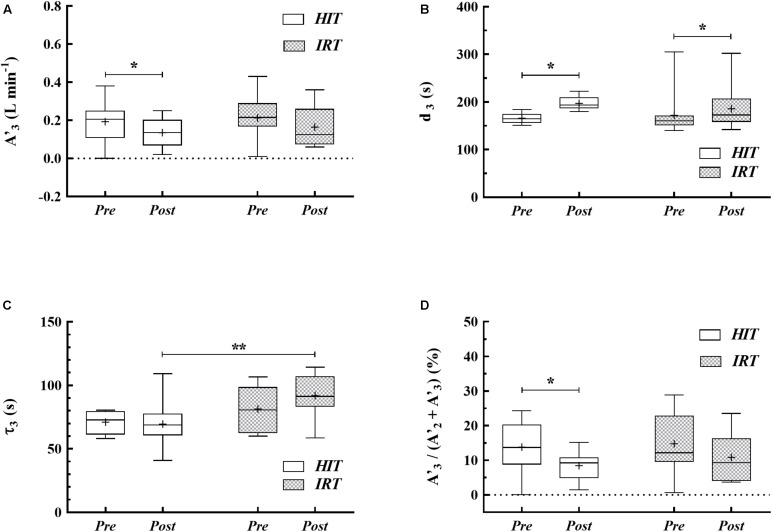
Box-whiskers graphs of the amplitude (*A*′_3_) of the *O*_2_ uptake at the end of exercise **(A)**, time delay **(B)** and slow phase time constant **(C)** of the 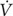
*O*_2sc_ and relative contribution of *A*′_3_ to the overall 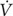
*O*_2_ response **(D)** at *Pre HIT*, *Post HIT*, *Pre IRT*, and *Post IRT*. Horizontal line marks the median of the data distribution, box extends from the 25th to the 75th percentiles; whiskers extend down to the 5th percentile and up to the 95th percentile; the cross represents the average. ^∗^*P* < 0.05; ^∗∗^*P* < 0.01.

After *HIT A*′_3_ was significantly smaller (*P* = 0.045; *d* = 0.65; 95% *CI*_Diff_: −0.01 L min^−1^/−0.11 L min^−1^) and *d_3_* was larger (*P* = 0.001; *d* = 1.55; 95% *CI*_Diff_: 19.6 s/43.1 s) than at *Pre HIT* (**Table 1** and **Figures 3A,B**). The relative contribution of the slow component to the overall 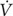
*O*_2_ response [*A*′_3_*/(A*′_2_ + *A*′_3_)] (**Figure 3D**) was significantly smaller at *Post HIT* than at *Pre HIT* (*P* = 0.018; *d* = 0.80; 95% *CI*_Diff_: −1.4%/−9.2%). Also, *IRT* affected *d_3_* (**Figure 3B**), as it was longer at *Post IRT* than at *Pre IRT* (*P* = 0.039; *d* = 0.67; 95% *CI*_Diff_: 2.0 s/25.3 s). In addition, a significant interaction between training types and time on *d_3_* was observed (*P* = 0.022; *d* = 0.87; 95% *CI*_Diff_: 6.1 s/39.5 s), indicating that *HIT* induced a more marked effect than *IRT* on *d_3_* (**Figure 3B**). Finally, τ_3_ was significantly greater at *Post IRT* than at *Post HIT* (*P* = 0.003; *d* = 1.107; 95% *CI*_Diff_: 10.7 s/34.1 s) (**Figure 3C**).

Training did not affect the parameters describing the increase in *HHb* at the onset of *HiEx* exercise (**Table 2**): no changes in *A*_1_, *d*_1_, τ_1_, and *MRT* were observed after either *HIT* or *IRT* as compared with the pre-training conditions.

**Table 2:** Mean values (SD) of the parameters describing [*HHb*] kinetics at the onset of *CWR* exercise of heavy intensity (*HiEx*) together with the values of [*HHb*] and [*Hb*_tot_] after 120 s of exercise and at the end of exercise.

Parameter				Training			
				HIT		IRT	
	*P*_b_; $\eta_{b}^{2}$	*P*_w_; $\eta_{w}^{2}$	*P*_int_;$\eta_{\mathrm{inter}}^{2}$	Pre	Post	Pre	Post
*[Hb*_tot_*]*_120_ (μM)	0.052; 0.300	0.814; 0.005	0.772; 0.008	4.5 (3.4)	3.8 (5.6)	6.8 (6.3)	7.0 (6.0)
*[Hb*_tot_*]*_end_ (μM)	0.016; 0.425	0.888; 0.002	0.917; 0.504	4.3 (2.3)	4.3 (6.0)	9.5^∗^ (7.5)	9.1 (6.5)
*[HHb]*_A1_ (s)	0.051; 0.307	0.246; 0.122	0.995; 0.000	8.3 (4.9)	9.8 (6.8)	12.2 (8.0)	13.7 (9.9)
*[HHb]*_d1_ (s)	0.572; 0.029	0.966; 0.000	0.199; 0.144	5.9 (1.0)	6.7 (3.2)	6.4 (1.4)	5.7 (2.6)
*[HHb]*_τ 1_ (s)	0.132; 0.188	0.731; 0.011	0.324; 0.091	4.5 (1.1)	4.4 (0.9)	3.9 (1.2)	4.3 (1.0)
95% *IC [HHb]*_τ 1_ (s)				3.1–5.6	3.2–5.8	2.2–6.0	3.6–5.1
*[HHb]*_MRT_ (s)	0.135; 0.191	0.821; 0.005	0.439; 0.055	10.4 (1.3)	11.1 (3.2)	10.3 (1.9)	10.0 (3.3)
*[HHb]*_120_ (μM)	0.091; 0.237	0.208; 0.140	0.627; 0.22	5.4 (3.2)	7.3 (6.3)	7.5 (4.6)	8.5 (5.9)
*[HHb]*_end_ (μM)	0.042; 0.324	0.121; 0.204	0.590; 0.027	5.4 (3.0)	6.4 (4.9)	6.9 (4.1)	9.0 (6.1)
Δ*[HHb]*/Δ 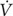 *O*_2_ (μM L^−1^ min^−1^)	0.078; 0.255	0.425; 0.060	0.922; 0.001	5.6 (3.1)	6.2 (3.7)	8.0 (5.3)	8.5 (6.2)

*Δ[HHb]/Δ
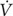
O_2_ ratio is also reported. The table reports also the mean of the 2.5% and 97.5% percentiles of the 95% confidence interval for τ_1_ and the P-values of the ANOVA analysis together with the values of the corresponding partial squared correlation factors. For the meaning of the symbols, please refer to the text. ^∗^Significantly different from Pre HIT.*

## Discussion

We investigated the effects of *HIT* and *IRT* on 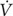
*O*_2_ kinetics during *CWR HiEx* exercise performed at the same absolute *WR* before and after training in a group of healthy, moderately active elderly men.

Post-intervention assessment after 8 weeks of *HIT* mainly showed:

(i) an increase in 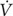
*O*_2 max_ and an improvement of *RCP;*
(ii) an increase in *Vol* and *CSA* of the quadriceps without a parallel increment of muscular strength;
(iii) a decrease in the amplitude *A*′_3_ of the slow component of 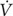
*O*_2_ kinetics assessed during *HiEx* together with a prolonged *d*_3_;

Post-intervention assessment after 8 weeks of *IRT* showed:

(i) a decrease in the functional gain of the primary phase of 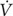
*O*_2_ kinetics during *ModEx*;
(ii) an increase in muscular *Vol* and *CSA* in parallel with a significant increment in muscular strength;
(iii) a deceleration in the primary component of 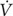
*O*_2_ kinetics with an increased τ_2_ during *HiEx*;
(iv) a significant increase of *d_3_* of 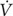
*O*_2sc_ In addition, the longer τ_3_ after *IRT* made the kinetics of 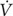
*O*_2sc_ significantly slower than the one after *HIT*.

### Maximal Oxygen Uptake and Gas Exchange Thresholds

Several studies have demonstrated the efficacy of *HIT* in increasing 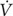
*O*_2 max_ in different populations (Kohrt et al., 1991; Bruseghini et al., 2015). Our results are in line with the findings that 8–12 weeks of interval training can induce a significant increase in 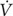
*O*_2 max_ in elderly subjects (Lepretre et al., 2009). *HIT* induced also a significant improvement in *RCP*. A comparable trend of the positive effects of *HIT* was found at intensities corresponding to the ventilatory threshold in elderly subjects (Pogliaghi et al., 2006).

### Muscle Morphology and Strength

The increases of *CSA* (plus 4.3% ± 3.6) and *Vol* (plus 5.6% ± 3.6) found after *HIT* are in agreement with previous findings (Sillanpää et al., 2008; Harber et al., 2012) that showed a significant increase (+6%) of the quadriceps muscle volume in elderly men after 12 weeks of aerobic training paralleled by the increase of *CSA* of myosin heavy chain type I (MHCI) fibers and a higher muscular thickness of *vastus lateralis* and *intermedius* after endurance training in older adults. *IRT* was followed by significant increases of *CSA* and *Vol* (plus 4.2% ± 4.4 and plus 4.9% ± 7.0). This confirms the results obtained in untrained elderly subjects in other occasions (Lee et al., 2008; Shunkert et al., 2008).

*IRT* was paralleled by a significant increase in the torque produced by the limb extensors. Therefore, we may also reasonably assume that, at *Post IRT*, our subjects were able to pedal at the same *WR* recruiting a smaller number of MUs. Indeed, from the individual *WR* and the isokinetic torque values we can calculate that the average torque maintained by the subject during *HiEx* exercise, when expressed as a percent of *T*_k_, significantly decreased from *Pre IRT* to *Post IRT*: *P* = 0.008, *CI* of the difference 1.3/−0.5 N m for *T*_k_ 60° s^−1^; *P* = 0.012, *CI* of the difference 1.3/−0.25 N m for *T*_k_ 120° s^−1^.

### Response to CWR of Moderate Intensity Exercise

The significant decrease in *G* after *IRT* reflected a decrease in the *O*_2_ cost of exercise and translated into a small, albeit significant, increase of 5% in work efficiency, η (*Pre IRT* η 22.9% vs. *Post IRT* η 24.0%; *P* = 0.041; 95% *CI*_Diff_: 0.1%/2.0%). This is consistent with previous findings of a significant decrease in the amplitude of the primary phase in cycling (Zoladz et al., 2012) after strength training. Accordingly, strength training results in an improved delta η (Bastiaans et al., 2001) and work η (Sunde et al., 2010) in cycling. The improvement in η found after *IRT* remains difficult to explain, though. One may surmise that, by increasing the absolute strength of the muscles involved in cycling, the subjects were pedaling against the same workload recruiting a smaller number of less efficient Type II fibers, wherefrom a smaller *G* and a larger η derived.

### Response to CWR During Heavy-Intensity Exercise

Endurance training is followed by a substantial reduction of 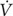
*O*_2sc_ (Casaburi et al., 1987; Womack et al., 1995). In addition, the contribution of 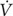
*O*_2sc_ to the overall 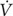
*O*_2_ response has been related to the % of Type I fibers, which have been described to have a greater metabolic stability (Hochacka and McClelland, 1997). Therefore, an increase of the % of Type I fibers at *Post HIT* may have led to improved metabolic stability (Zoladz et al., 2006), attenuated the drop in intramuscular *pH* and *[PCr]* and decreased 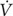
*O*_2sc_ (Jones et al., 2007).

In addition to these mechanisms directly linked to the plausible phenotypical shift in muscle fiber populations, also mechanisms intrinsic to each single fiber may be responsible for the observed decrease of 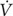
*O*_2sc_ after *HIT*. A recent study (Zoladz et al., 2016) showed that endurance training in rats induced a temperature dependent enhancement of mitochondrial oxidative phosphorylation and a significant drop of mitochondrial uncoupling. Therefore, the decrease of *O*_2_ cost for oxidative *ATP* production in each recruited muscle fiber may have substantially potentiated the effect of endurance training on 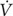
*O*_2sc_.

It has been also shown that 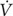
*O*_2sc_ is modulated by manipulations of *O*_2_ delivery (Poole and Jones, 2012). *HIT* may improve *O*_2_ availability and induce a better matching between *O*_2_ delivery and utilization (Murias et al., 2010a,b). This may have a positive impact on 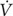
*O*_2sc_ in the elderly in whom metabolic vasodilatation is impaired (Poole et al., 2003) and a mismatch of local *O*_2_ delivery to *O*_2_ muscular consumption is present (Murias et al., 2010a,b). However, the obtained results do not support this conclusion, as training did not modify any of the indexes that characterize *HHb* response during *HiEx* exercise. In particular, the primary time constant *[HHb]_τ__1_* was not affected: a constant τ_1_ would suggest a proportionally similar increase of the speeds of adjustment of local *O*_2_ delivery and muscular *O*_2_ uptake in the primary phase of 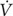
*O*_2_ kinetics during *HiEx*. This conclusion is somehow strengthened by the observation that *[HHb]*_end_ was not modified in presence of a lower *A*′_3_ of *O*_2_ uptake response.

The primary phase of τ_2_ of 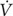
*O*_2_ kinetics during *HiEx* was decelerated after *IRT*. In analogy with *ModEx*, the primary component in *HiEx* exercise is thought to increase exponentially without other changes (Poole and Jones, 2012). However, because the statistical estimate of τ_2_ during *HiEx* is often based on a limited number of data, this unavoidable drawback may produce uncertain and unreliable values of τ_2_. Besides these methodological problems, specific physiological adaptations induced by *IRT* may have contributed to the increase of τ_2_. A constant *[HHb]_τ_* helps us infer that local *O*_2_ delivery response and muscular *O*_2_ utilization changed proportionally after training. Therefore, a substantial defect in *O*_2_ availability may be still present after *IRT*.

The changes induced by *IRT* on 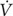
*O*_2sc_ are somehow ambiguous: the amplitude of 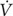
*O*_2sc_, either in absolute or relative terms, turned out to be unaffected by *IRT*, but 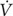
*O*_2sc_ appeared later and developed more slowly than at *Post HIT*.

The mechanism underpinning the delay in the appearance of 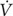
*O*_2sc_ after *HIT* and *IRT* are of different origin. We first underline that *IRT* training was effective in increasing the strength of muscles involved in pedaling (see the section “Results”). Therefore, we can suggest that the pedaling subjects after *IRT* were utilizing a lower percentage of their maximal voluntary force at the same *WR* and that they were recruiting a smaller number of Type II MUs. Should this be true, the diminished recruitment of these MUs would result in a slower development of 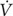
*O*_2sc_, as the utilized Type I muscle fibers are less liable to develop fatigue and their metabolic features make them less prone to cause 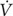
*O*_2sc_. However, this explanation does not clarify whether the main cause of 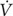
*O*_2sc_ resides in *intensive* mechanisms, i.e., the progressive decay of the efficiency of the already recruited MUs, or, rather, it may be ascribed to an *extensive* process, i.e., the progressive recruitment of less efficient Type II fibers. It is worth noting, however, that the net decrease of *d_3_* observed after *IRT* was positively correlated with the net increase of *knee torque* (*P* = 0.046, *r* = 0.60). Conversely, the two variables were not correlated in the case of *HIT* (*P* = 0.316, *r* = −0.32).

Also, after IRT, we were not able to find any significant changes of muscular oxygenation and of the indexes that describe amelioration of local peripheral perfusion. This might suggest that the impairment of local *O*_2_ delivery was not the main cause of 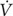
*O*_2sc_, at least in this specific population of subjects.

### Points of Strength and Weakness of the Study

We compared for the first time the effects of *HIT* and of *IRT* on the dynamic response of pulmonary 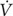
*O*_2_ and muscular oxygenation during *HiEx* exercise in healthy, untrained elderly men.

However, a few methodological limitations should be mentioned. The experimental design was not counterbalanced for reasons of feasibility.

We did not evaluate the changes in muscle fiber expression during the two training interventions. Since the size of 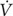
*O*_2sc_ has been positively related to the percentage of Type II fibers (Poole and Jones, 2012), a strong correlation between the observed changes in the slow component and the changes in the phenotypical expression of the trained muscles would have strengthened the hypothesis of a muscular origin of the slow component.

We did not evaluate the possible changes in neuromuscular activation induced by the two training modalities during *ModEx* and *HiEx*. Comparison of the differences in recruitment patterns would have helped to strengthen or reject our working hypothesis on the role of Type II motor units involvement in the genesis of 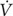
*O*_2sc_ after *IRT*.

*HHb* signal mainly reflects the fractional *O*_2_ extraction of the interrogated zone of the muscle resulting from the dynamic balance between muscular *O*_2_ uptake and local *O*_2_ delivery and *HHb* signal reflects changes in oxygenation mainly in the capillaries of the explored muscle volume (Grassi and Quaresima, 2016). However, the assessment of *HHb* obtained only from the surface of the *vastus lateralis* may be a substantial limitation to our analysis, since some spatial heterogeneity in terms of muscle oxygenation in an exercising muscle seems to exist (Koga et al., 2007). Nonetheless, using skin landmarks to accurately place the NIRS probe in the same site before all experiments minimized possible problems due to spatial inhomogeneity.

Finally, the study aimed to investigate the effects of training in a particular population of subjects, i.e., elderly healthy volunteers who may have larger strength deficits than young, active adults. Therefore, the meaning and the applicability of the results obtained in this study may be extended with some caution to other classes of subjects.

## Conclusion

The amplitude of 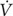
*O*_2sc_ during *HiEx* was substantially smaller after *HIT* than before, but its decrease was not correlated with an improvement in the *O*_2_ delivery-to-utilization ratio of the exercising muscles. This suggests that suboptimal local *O*_2_ delivery was not a possible factor contributing to 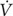
*O*_2sc_ in the elderly, whereas the improved metabolic stability induced by *HIT* was likely able to induce beneficial effect on 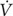
*O*_2sc_.

*IRT*, by increasing muscle strength, resulted in a delayed appearance of 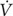
*O*_2sc_ during *HiEx* because of a possible larger contribution of Type I fibers to a motor task of identical absolute intensity.

### Perspectives

The results obtained in the present investigation may have practical applications. First, the association between a larger exercise economy and the delayed appearance of 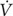
*O*_2sc_ found after *IRT* may be of interest, as it suggests that strength training should be included in the usual training programs of elderly people to improve exercise economy and resistance to fatigue (Beattie et al., 2014). Second, they prompt the investigators to better characterize the changes in neuromuscular activation and MUs recruitment induced by the two training modalities during *HiEx* performed at the same *WR*. This should be done in parallel with the invasive evaluation of the changes in muscle fiber expression induced by training interventions.

## Author Contributions

CC, PB, ET, FS, and EC planned the study. CC, ET, PB, EO, AP, RPM, SP, and EC collected and analyzed the data. CC, PB, and ET wrote the manuscript. CC, PB, ET, EC, and RPM revised the manuscript.

## Conflict of Interest Statement

The authors declare that the research was conducted in the absence of any commercial or financial relationships that could be construed as a potential conflict of interest.
